# Development and Characterization of a Tacrolimus/Hydroxypropyl-β-Cyclodextrin Eye Drop

**DOI:** 10.3390/pharmaceutics13020149

**Published:** 2021-01-23

**Authors:** Xurxo García-Otero, Victoria Díaz-Tomé, Rubén Varela-Fernández, Manuel Martín-Pastor, Miguel González-Barcia, José Blanco-Méndez, Cristina Mondelo-García, Maria A. Bermudez, Francisco Gonzalez, Pablo Aguiar, Anxo Fernández-Ferreiro, Francisco J. Otero-Espinar

**Affiliations:** 1 Pharmacology, Pharmacy and Pharmaceutical Technology Department, Faculty of Pharmacy, University of Santiago de Compostela (USC), 15705 Santiago de Compostela, Spain; xurxo.garcia@rai.usc.es (X.G.-O.); victoria.diaz@rai.usc.es (V.D.-T.); ruben.varela.fernandez@rai.usc.es (R.V.-F.); jose.blanco.mendez@usc.es (J.B.-M.); 2 Molecular Imaging Group, University Clinical Hospital, Health Research Institute of Santiago de Compostela (IDIS), 15706 Santiago de Compostela, Spain; 3 Clinical Pharmacology Group, University Clinical Hospital, Health Research Institute of Santiago de Compostela (IDIS), 15706 Santiago de Compostela, Spain; miguel.gonzalez.barcia@sergas.es (M.G.-B.); cristina.mondelo.garcia@sergas.es (C.M.-G.); 4 Clinical Neurosciences Group, University Clinical Hospital, Health Research Institute of Santiago de Compostela (IDIS), 15706 Santiago de Compostela, Spain; 5 Nuclear Magnetic Resonance Unit, Research Infrastructures Area, University of Santiago de Compostela (USC), 15782 Santiago de Compostela, Spain; manuel.martin@usc.es; 6 Paraquasil Group, University Clinical Hospital, Health Research Institute of Santiago de Compostela (IDIS), 15706 Santiago de Compostela, Spain; 7 Physiology Department–CIMUS, University of Santiago de Compostela (USC), 15782 Santiago de Compostela, Spain; maria.alvarez.bermudez@udc.es; 8 Ophthalmology Department, Clinical University Hospital Santiago de Compostela (SERGAS), 15706 Santiago de Compostela, Spain; francisco.gonzalez@usc.es; 9 Department of Surgery and Medical-Surgical Specialties and CIMUS, University of Santiago de Compostela (USC), 15782 Santiago de Compostela, Spain

**Keywords:** tacrolimus, hydroxypropyl-β-cyclodextrin, topical ophthalmic administration, eye drops, uveitis, PET/CT imaging

## Abstract

Uveitis is a vision inflammatory disorder with a high prevalence in developing countries. Currently, marketed treatments remain limited and reformulation is usually performed to obtain a tacrolimus eye drop as a therapeutic alternative in corticosteroid-refractory eye disease. The aim of this work was to develop a mucoadhesive, non-toxic and stable topical ophthalmic formulation that can be safely prepared in hospital pharmacy departments. Four different ophthalmic formulations were prepared based on the tacrolimus/hydroxypropyl-β-cyclodextrin (HPβCD) inclusion complexes’ formation. Phase solubility diagrams, Nuclear Magnetic Resonance (NMR) and molecular modeling studies showed the formation of 1:1 and 1:2 tacrolimus/HPβCD inclusion complexes, being possible to obtain a 0.02% (*w/v*) tacrolimus concentration by using 40% (*w/v*) HPβCD aqueous solutions. Formulations also showed good ophthalmic properties in terms of pH, osmolality and safety. Stability studies proved these formulations to be stable for at least 3 months in refrigeration. Ex vivo bioadhesion and in vivo ocular permanence showed good mucoadhesive properties with higher ocular permanence compared to the reference pharmacy compounding used in clinical settings (*t_1/2_* of 86.2 min for the eyedrop elaborated with 40% (*w/v*) HPβCD and Liquifilm^®^ versus 46.3 min for the reference formulation). Thus, these novel eye drops present high potential as a safe alternative for uveitis treatment, as well as a versatile composition to include new drugs intended for topical ophthalmic administration.

## 1. Introduction

Uveitis is a sight-threatening inflammatory disorder that affects a wide range of ages in the world population, being the main cause of 5–20% of blindness in Europe and United States and over 25% in developing countries [1,2]. This affection can lead to other types of complications, including cataract, increased intraocular pressure (IOP), macular edema (ME) or glaucoma, compromising visual loss [3,4,5]. Therefore, it causes a great clinical and socioeconomic impact on the life quality of patients [6,7,8] and early diagnosis and treatment are important to prevent complications.

Uveitis is a recurrent and common ophthalmic disease that has an idiopathic etiology, caused by a complication of an infection or associated with a systemic disease (infectious or autoimmune disorder) [3,9]. The pathogenesis of uveitis is often caused by an autoimmune response. Inflammatory cytokines promote the activation of T cells and trigger recruitment of large numbers of circulation inflammatory leukocytes into the eye. This process may cause irreversible tissue damage and visual impairment. Topical corticosteroids constitute the first therapeutic line to treat the disease, but remarkable adverse effects can appear due to continuous treatment with these drugs. The use of immunosuppressants in uveitis is indicated in corticosteroid-refractory eye disease or after systemic side effects’ appearance.

Tacrolimus is a macrolide with a high molecular weight (804.02 g/mol) isolated from *Streptomyces tsukubaensis*, with a great immunosuppressive activity (100 times more potent than cyclosporine A) [10,11] that inhibits T cell proliferation and suppresses the release of inflammatory cytokines; it can theoretically be used to reduce inflammatory activity in uveitis patients [3]. This drug has been used in different ocular diseases including corneal graft rejection [12,13,14], vernal keratoconjunctivitis (VKC) [15,16,17], dry eye [11,18], uveitis [5,19,20], scleritis [21,22] or graft-versus-host disease [23,24,25], among others. Additionally, clinical studies have shown tacrolimus’ high effectivity compared to other immunosuppressants such as cyclosporine, at lower concentrations [26].

Nowadays, tacrolimus eye drops are not marketed and all its use rests in the elaboration in hospital pharmacy departments (HPDs). The preparation of ophthalmic tacrolimus formulations is limited by its poor water solubility (1–12 μg/mL) [27,28] due to the hydrolytic mechanism [29]. For this reason, 0.03% (*w/v*) tacrolimus eye drops are being obtained by reformulation from intravenous drug presentation (Prograf^®^) [15]. However, in this formulation, tacrolimus is solubilized in ethanol, containing irritating compounds that usually cause discomfort and unpleasantness to the patient. Based on these statements, it would be interesting to design new topical ophthalmic formulations with the lowest possible toxic potential and better tolerability. Several types of tacrolimus formulations such as niosomes [30], nanoemulsions [5], microspheres [31], nanocapsules [32], micelles [33], emulsions [34] or liposomes [35] have also been described by other authors. Nonetheless, if these are not synthesized to be marketed, their elaboration in HPDs will be complicated.

The need to increase the solubility and stability of the drug becomes a task of extreme necessity. There are several types of β-cyclodextrin (βCD) derivatives, although most of them have not been authorized or there are not enough preclinical studies to support their use at the topical ophthalmic level [36]. Some β-cyclodextrins have been used by other authors as complexing agents with several drugs [27,37,38]. The oligosaccharide 2-hydroxypropyl-β-cyclodextrin (HPβCD) is a cyclic oligosaccharide formed by seven units of α-1,4-linked glucose and a hydroxypropylated group, with a lipophilic central cavity and a hydrophilic outer surface. The rigid doughnut-shaped structure makes it a natural complexing agent, being able to form inclusion complexes with several drugs, where their structure (or part of it) may fit in the cyclodextrin cavity [39]. The complexation between drugs and cyclodextrin affects many drugs’ physicochemical properties, including their chemical stability and aqueous solubility [39]. Therefore, this hydrophilic cyclodextrin derivative can form highly water-soluble complexes with lipophilic drugs, as it happens with tacrolimus. There are few reports on the use of cyclodextrins to improve the pharmaceutical characteristics of tacrolimus [37]. However, HPβCD has been chosen in this work due to the fact that, according to the European Medicines Agency (EMA), it is the safest and most appropriated cyclodextrin for topical ophthalmic administration, proving that it was not toxic [40]; this fact becomes important when transferring research to the clinic. Hence, there is already a commercialized formulation (Indocollyre^®^ 0.1% ophthalmic solution) complexing HPβCD and indomethacin [41]. The improvement of aqueous tacrolimus eye drops is still a challenge because of its low chemical stability and solubility in particular [27].

The aim of this work was based on the design and development of different topical ophthalmic formulations containing tacrolimus as an alternative to the reformulated Prograf^®^ intravenous solution (REF). The characterization of these tacrolimus-loaded ophthalmic formulations incorporating the improvements that cyclodextrin (HPβCD) properties can provide in terms of tacrolimus solubility and stability in aqueous solution was also carried out. Cyclodextrin properties may also improve the mucoadhesive characteristics of the formulations, leading to a retention time increase on the ocular surface. In addition, the use of new molecular imaging techniques such as positron emission tomography/computed tomography imaging (PET/CT imaging) was incorporated into the present study in order to better understand the in vivo formulations’ permanence on the corneal surface. On the other hand, the evaluation of the eye drops’ safety was necessary to verify that there were no corneal surface alterations. Based on this background, the novelty of this work relies on the obtention of a consistent preclinical base for a new safe, stable and bioadhesive tacrolimus eye drop designed for appropriate preparation by HPDs. Besides, additional goals were also pre-established such as ease of preparation, scalability from the laboratory scale to HPDs and patient comfort improvement.

## 2. Materials and Methods

Tacrolimus powder was acquired from Guinama^®^ S.L.U. (La Pobla de Vallbona, Spain), 2-hydroxypropyl-β-cyclodextrin Kleptose^®^ HPB (HPβCD; M_W_ = 1399 Da, substitution degree = 0.65 molar ratio) was provided from Roquette Laisa S.A.^®^ (Valencia, Spain), Liquifilm^®^ was purchased from Allergan^®^ Pharmaceuticals Ireland (Mayo, Ireland), Balanced Salt Solution (BSS^®^) was acquired from Alcon^®^ laboratories (Fort Worth, TX, USA) and Prograf^®^ (5 mg/mL, ampoules) was purchased from Astellas Pharma S.A.^®^ (Madrid, Spain). Ultrapure water MilliQ^®^ (Millipore Iberica; Madrid, Spain) was used throughout the whole work. All other chemicals and reagents were of the highest purity grade commercially available.

### 2.1. Tacrolimus/HPβCD Solubilization Study

#### 2.1.1. Phase Solubility Diagram

A phase solubility diagram was used to estimate the stability constant (*K*) of the tacrolimus/HPβCD inclusion complex. The solubility measurements were carried out following the previous work of our research group, described by Anguiano-Igea et al. [42]. This protocol was adjusted to the Higuchi and Connors phase solubility model [43]. A solubility study was carried out by adding an excess amount of the tacrolimus powder to MilliQ^®^ aqueous solutions containing increasing concentrations of HPβCD (from 0 to 400 mg/mL). The vials containing these suspensions were then shaken in a VWR incubated mini-shaker (VWR International, Radnor, PA, USA) at 25 °C and 250 rpm for 7 days until reaching an equilibrium. Subsequently, each aliquot was centrifuged for 30 min at 15,300 rcf (Centrifuge 5804 R; Eppendorf^®^, Hamburg, Germany) and 100 µL of the supernatant was diluted with 400 μL of MilliQ^®^ water (1:5 dilution) prior to measurement. Each HPβCD concentration was assayed in quintuplicate.

Tacrolimus concentration was determined for each sample using an HPLC system (Agilent 1260 series; Agilent Technologies^®^, Santa Clara, CA, USA) equipped with a Diode Array Detector HS, a solvent delivery quaternary pump system, 400 bar maximum pressure and an autosampler. The software model OpenLAB CDS 3D UV (PDA) was used for the data processing. The analysis was performed under an isocratic method. The column used was a Poroshell 120, EC-C18 (4.6 × 100 mm, 4 µm) and at a temperature of 60 °C. The mobile phase was water-acetonitrile (35:65 (*v/v*)) using a 1.5 mL.min^−1^ flow rate. A 210-nm wavelength was employed for the tacrolimus quantification. The volume of the injected sample was 10 μL and the retention time was 3 min. The analytical method was validated according to International Conference on Harmonization (ICH) guideline recommendations [44] and the mathematical adjustments were made in GraphPad Prism^®^ 8 v.8.2.1 software (GraphPad Software, San Diego, CA, USA, 2019).

A_P_ phase solubility types are usually observed when a drug molecule forms a complex with more than one CD molecule, assuming a consecutive complexation. A quadratic model equation allows the estimation of both stability constants (*K_1:1_* and *K_1:2_*). The value of *K_1:2_* is often lower than that of *K_1:1_*.

These constants values were calculated using the following Equation (1):

*S* = *S_0_* +(*K_1:1_*·*S_0_* + *K_1:1_*·*K_1:2_*·*S_0_*·[HPβCD])·[HPβCD], (1)

where *S* is the total solubility, *S_0_* is the free drug solubility and *K_1:1_* and *K_1:2_* are the stability constants of the complex tacrolimus/HPβCD. *K_1:1_* and *K_1:2_* values were calculated by non-linear regression using GraphPad Prism^®^ 8 v.8.2.1 software.

#### 2.1.2. NMR Studies and Molecular Modeling

NMR experiments were conducted at two different temperature conditions, 278 and 298 K, on a Bruker NEO 17.6 T spectrometer (proton resonance 750 MHz) (Billerica, MA, USA), equipped with a ^1^H/^13^C/^15^N triple resonance PA-TXI probe with a deuterium lock channel and a shielded Pulse Field Gradient (PFG) z-gradient. The spectrometer control software was TopSpin 4.0 (Billerica, MA, USA, 2020). The reported chemical shifts are referenced to the lock deuterium solvent. Spectra were processed and analyzed using Mestrenova v14.0 software (Mestrelab Research S.L., Santiago de Compostela, Spain, 2019).

One-dimensional saturation transfer difference ^1^H spectra (STD) [45] were measured. The experiment consisted of a selective saturation pulse train, a WET selective solvent suppression module and a 90-degree hard-pulse followed by *fid (free induction decay)* acquisition. The selective saturation consisted of a train of soft Gaussian-shaped pulses of 50-ms duration with a 1-ms interpulse delay. The selective saturation was applied during 2 s at a specific frequency of the ^1^H spectrum, covering a region of the spectrum of ± 125 Hz around the chosen frequency (i.e., ± 0.17 ppm in a 750 spectrometer). The STD^off^ saturation was applied at 20 ppm. The STD^on^ saturation was applied at the frequency of one specific signal of tacrolimus separated by more than 300 Hz from any signals of the HPβCD. The STD^on^ and STD^off^ scans were measured in alternate scans and subtracted by the phase cycling, providing the subtracted STD^off-on^ spectra. Three STD spectra were obtained by STD^on^ saturation of the tacrolimus signals at 6.30, 6.12 and 2.12 ppm, respectively. Each spectrum was acquired in 15 min with 128 scans and a 6.75-s total scan duration consisting of a 2-s pre-scan d_1_, a 2-s STD saturation time and a 2.75-s *fid* acquisition.

Molecular modeling was also performed to have an orientation of which is the most likely interaction between tacrolimus and HPβCD molecules using an MM+ force field in HyperChem^®^. The HPβCD used in this work (Kleptose^®^ HPB) was a mixture of hydroxypropylated-β-CDs with a substitution mean degree of 0.65 (0.58–0.68; a mean of 4.2 hydroxypropyl groups per cyclodextrin molecule). An HPβCD molecule type with an average of four hydroxypropyl groups per native CD unit was used to obtain the molecular model.

#### 2.1.3. Vehicle Solubility Study

In addition to conducting a MilliQ^®^ water solubility study, it was studied whether the tacrolimus solubility varied in different media in which the final tacrolimus eye drops could be formulated. The tacrolimus solubility was tested in two different vehicles besides MilliQ^®^ water, these being BSS^®^ and Liquifilm^®^.

Three HPβCD proportions (20%, 30% and 40% (*w/v*)) were used sixfold with each vehicle and a two-way ANOVA was subsequently applied to compare the formulations’ solubility and to check for significant differences among them. This assay was carried out as a way to select the formulations to be tested for further analysis.

### 2.2. Formulation Preparation Procedure

The 2-hydroxypropyl-β-cyclodextrin (HPβCD) solutions were prepared by dissolving them in the two vehicles studied in the previous assay (BSS^®^ and Liquifilm^®^); these were selected because they are ophthalmic vehicles that are frequently used in HPDs for eye drop formulation. BSS^®^ is an isotonic salt solution for use in irrigating eye tissues, with pH 7.5 and a ≈300 mOsm/kg osmolality [46]. Furthermore, Liquifilm^®^ is an artificial eye tear that contains 1.4% polyvinyl alcohol (PVA) and a benzalkonium chloride preservative as well as different salts [47].

Based on the vehicle solubility study results (see Section 2.1. Tacrolimus solubilization with HPβCD results), the HPβCD and tacrolimus concentrations were chosen. The formulations were labeled as TBS 20 (20% (*w/v*) HPβCD and 0.01% (*w/v*) of tacrolimus in BSS^®^), TBS 40 (40% (*w/v*) HPβCD and 0.02% (*w/v*) of tacrolimus in BSS^®^), TLI 20 (20% (*w/v*) HPβCD and 0.01% (*w/v*) of tacrolimus in Liquifilm^®^) and TLI 40 (40% (*w/v*) HPβCD and 0.02% (*w/v*) of tacrolimus in Liquifilm^®^) (see Table 1). Once the cyclodextrin was dissolved, tacrolimus powder was added under magnetic stirring (>750 rpm) until all the tacrolimus powder was dissolved.

The reference formulation was prepared just as it is formulated in an HPD; a mixture of 0.03% (*w/v*) tacrolimus in Liquifilm^®^ was prepared by a Prograf^®^ (5 mg/mL) intravenous ampoule dilution as it was done in previous work [15], instead of using the tacrolimus powder. All formulations were kept under refrigeration conditions (4 ± 2 °C) to avoid degradation processes of the drug when they were prepared. Tacrolimus concentrations used in clinical practice are very variable (0.005% to 0.1% range) [11,17,48,49]; it should be noted that the selection of the designated tacrolimus concentration has been carried out on the basis of previous experimental studies (see Section 2.1.3. Vehicle solubility study).

### 2.3. Optimization Procedure: Tacrolimus Solubilization Time

The purpose of this assay was to know the required tacrolimus solubilization time in the eye drops so that when the formulation has to be replicated in an HPD, the drug will not be removed when performing the sterilizing filtration, ensuring quality, final product efficacy and patient safety.

This study was designed to establish the minimum stirring time of the tacrolimus powder to achieve the desired concentration in the solution media. HPβCD dissolution was carried out in triplicate (10 mL per formulation), and the amount of tacrolimus powder was added once all the cyclodextrin was dissolved. The solutions were shaken in a Cimarec I multipoint magnetic stirrer (ThermoFisher^®^ Scientific; Waltham, MA, USA) at 900 rpm and 30 °C during the whole study period.

At predetermined times (24, 40, 48, 67, 72, 90 and 96 h), samples were collected without stopping the stirring, thus achieving the dissolution homogeneity. Samples were centrifuged (Centrifuge 5804 R; Eppendorf^®^, Germany) at 15,300 rcf and 25 °C for 30 min to remove any undissolved particles. Subsequently, 400 µL of the supernatant was taken to measure the tacrolimus concentration by HPLC (Agilent 1260 series; Agilent Technologies^®^, Santa Clara, CA, USA) with the method described above (see Section 2.1.1. Phase solubility diagram). The tacrolimus concentration was corrected in each measure according to the volume that was left (0.5 mL less in each sample). Resulting data were compared and a statistical analysis was performed using a two-way ANOVA to analyze the influence of the solubilization time and the composition of the formulations. The analysis was carried out using GraphPad Prism^®^ 8 v.8.2.1 software.

### 2.4. Physicochemical Characterization

#### 2.4.1. pH and Osmolality Determination

The osmolality measurements were performed with an OsmoSpecial 1 osmometer (Astori Tecnica^®^; Poncarale, Italy), while the pH was measured using a pH meter (HI5221 HANNA^®^, Italy) at 25 ± 0.5 °C. Each determination was carried out in triplicate.

#### 2.4.2. Surface Tension Determination

Surface tension at the surface-to-air interface was measured using the du Nöuy ring method [50]. A Lauda TD1 tensiometer (LAUDA Scientific GmbH^®^, Lauda-Königshofen, Germany) fitted with a platinum ring (2-cm diameter) was used to measure the surface tension of the tacrolimus formulations. A platinum du Nöuy ring was immersed into the liquid and then lifted to obtain tension values. The measurements were made at room temperature.

All glassware used for the surface tension measurements was washed with MilliQ^®^ water and then dried in a clean oven before use. The platinum du Nöuy ring was washed with alcoholic KOH, rinsed in MilliQ^®^ water and flamed until red-hot before each measurement. The determinations were carried out in triplicate.

#### 2.4.3. Squeezing Force Determination

The squeezing force test evaluates the force needed to dispense a drop from 5-mL polypropylene eyedropper bottles that are commonly used in HPDs. A comparison of the cyclodextrin formulations with each other and between the reference one was made in order to assess the presence or absence of significant differences depending on their composition.

**Figure 1:**
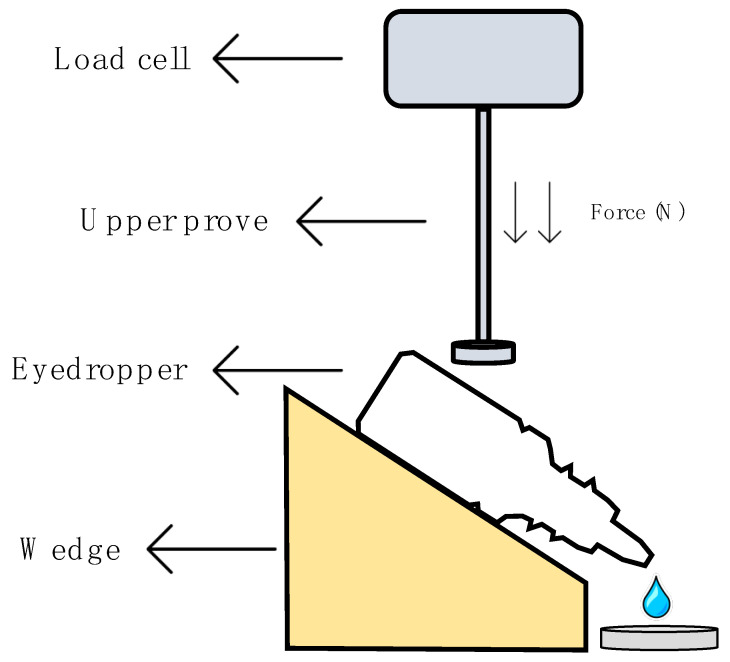
Scheme of the squeezing force determination.

This method was performed following the method established by Charles H. Cox, with minor modifications [51], in a Shimadzu^®^ texturometer (Kyoto, Japan). Figure 1 describes in detail the protocol followed to obtain the squeeze force measurements. The eyedropper bottle containing 5 mL of formulation was placed on a plate with a 45° inclination just below the load cell (1000 N maximum force). Test parameters were set after the method optimization; the upper probe moved down at a speed of 0.5 mm/s and an assessment of the required force to spill one drop of the formulation was performed. Five eyedropper bottles of each formulation were tested in quintuplicate (25 measurements per formulation). A one-way ANOVA was carried out to find out if there were significant differences. Tukey´s multiple comparisons test was subsequently performed in order to compare all formulations.

### 2.5. Corneal Mucoadhesion

**Figure 2:**
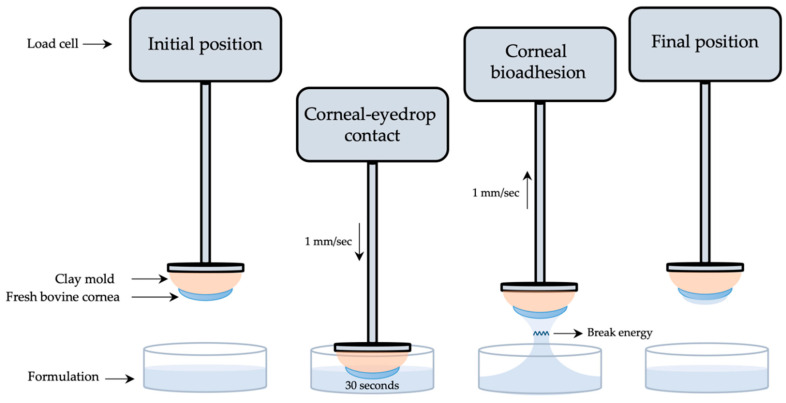
Scheme of the corneal mucoadhesion method.

Tacrolimus formulations’ mucoadhesion was measured following a method designed and developed by our research group using a Shimadzu^®^ texturometer [52]. Corneal bioadhesion was used to quantitatively determine the interaction between the formulation and the corneal bovine surface. As presented in Figure 2, fresh bovine corneas were adjusted to a cornea mold made of clay and subsequently fixed to a texturometer load cell (20 N maximum force). Formulations were dropped in a glass bottle (40 mm diameter, 20 mm height) and placed in the lower part of the analyzer.

Corneas were immersed 2 mm deep into the formulation at a 1 mm/s speed and force data (N) were obtained. Then, corneas were kept in touch with the formulation for 30 s, and just after returning to the starting point at a 1 mm/s speed, work (mJ) was measured. Mucoadhesion work was calculated from the area under the curve (AUC) of the force–displacement curve. All method parameters were previously studied and subsequently applied. Each measurement was carried out in triplicate.

#### 2.5.1. Bovine Corneal Opacity and Permeability Test (BCOP)

The Bovine Corneal Opacity and Permeability test (BCOP) is an organotypic assay used to identify potential ocular corrosives and severe irritants. This method was carried out using slaughterhouse materials (fresh bovine corneas), allowing the animal replacement compliance and avoiding the use of laboratory animals. This assay was founded on the method established by Tchao et al. [53] and adapted by Gautheron et al. [54], with minor modifications.

Freshly excised eyes were obtained from a local slaughterhouse and transported to the laboratory in optimal conditions. A macroscopic inspection of the eyes was performed, and only free-of-defects corneas were dissected and used for the test. Fresh corneas were mounted in Franz diffusion cells with the corneal epithelial surface facing upwards, dividing the two different chambers of the diffusion cell (donor and receptor). A thermostatic bath with a controlled temperature (37 ± 2 °C) and under stirring (100 rpm) during the whole assay was used to incubate all Franz diffusion cells.

Corneal opacity changes were measured by two different techniques, these being photometry and UV–Vis spectrophotometry. Photometry was assessed using a luxmeter (Gossen^®^ Mavolux 5032C USB; Nürnberg, Germany), where corneas were placed between two cylindrical supporting black holders (fabricated with polylactic acid filaments using a 3D printer, Witbox^®^ BQ, Madrid, Spain) and illuminated with a pipe light (Olympus^®^ Highlight 200, Tokyo, Japan) with fixed brightness values [55]. Corneal transparency was measured in transmittance values by UV–Vis spectrophotometry (from 200 to 800 nm), allowing the light to pass through the corneas, from the source to the receiver of the spectrophotometer (Cary 60 UV-Vis, Agilent Technologies^®^; Santa Clara, CA, USA). Each formulation was tested in triplicate.

The following protocol was performed for formulations’ addition and opacity measurements: (I) determination of the initial opacity values for freshly excised corneas by photometry and UV–Vis spectrophotometry; a corneal blank was made before photometry determination in order to remove basal light, while the cornea itself was used as a blank (800-nm wavelength) before spectrophotometry determination; (II) addition of 1 mL Phosphate-Buffered Saline (PBS) into the donor chamber of the vertical diffusion cells and cornea incubation were performed for 10 min, followed by opacity determination; (III) an amount of 1 mL of the formulation was then added to the upper chamber for 10 min, followed by its subtraction and further addition of PBS for 120 min; after this time, the opacity determination was repeated.

Once the opacity was measured, the same corneas were used to evaluate the corneal permeability changes, so 1 mL of 0.4% (*w/v*) fluorescein aqueous solution was added in the donor compartment, keeping in touch with the corneal epithelium side. After 90 min, the corneal permeability determination was evaluated by measuring the optical density (OD) in the medium (PBS) of the lower compartment at a 490-nm wavelength.

An in vitro irritation score (IVIS) can be calculated with the measurements of corneal opacity and permeability. The IVIS was determined by the following Equation (2):

IVIS = mean opacity value + (15 × mean permeability OD_490_ value), (2)

#### 2.5.2. Hen’s Egg Test on the Chorioallantoic Membrane (HETCAM)

The hen’s egg test on the chorioallantoic membrane (HETCAM) assay is one of the new organotypic models that allows the identification of irritative reactions, and it has become the international standard assay for acute eye irritation and corrosion (OECD TG 405, 2002; EC B.5). This assay was performed to evaluate possible acute ocular irritation caused by the present tacrolimus formulations.

Fertilized Broiler eggs (50–60 g weight) were obtained from the regional hatchery technology center and incubated for nine days in specific conditions (37 ± 0.5 °C and 65% ± 5% RH). The eggs were automatically rotated in an automatic rotational incubator every 2 h until the eighth day, where rotation was stopped, and the eggs were kept in the axial position during 24 h for the proper placement of the chorioallantoic membrane (CAM). The protocol used was adapted from the procedure described by Spielmann and Liebsch [56].

At the ninth day of incubation, embryonated eggs were opened by their upper part with a tiny drill (Dremel^®^, Madrid, Spain) and the inner membrane was moistened with 0.9% (*w/v*) NaCl aqueous solution so that it could be removed later. Briefly, 300 µL of 0.9% (*w/v*) NaCl solution (negative control), 0.1% (*w/v*) NaOH solution (positive control) and tacrolimus formulations (TBS 20, TLI 20, TBS 40, TLI 40 and REF) were directly instilled onto the CAM (2 eggs per compound). The membrane was observed over a 5-min period using an Olympus^®^ SZ61TR Stereomicroscope and Olympus^®^ CellSens Entry software.

Hemorrhage, lysis and coagulation of the CAM were measured; these reactions can be quantified through an irritation score (IS) following the Kalweit et al. criteria [57]. Based on this, they can be classified as follows: 0–0.9, no irritation; 1–4.9, slight irritation; 5–8.9, moderate irritation; and 9–21, severe irritation.

### 2.6. Stability Study

The stability study was only carried out with the formulations containing the greatest amount of drugs due to the fact that all the formulations have the same composition, the formulations with 40% (*w/v*) HPβCD being the most representative, as they have a greater amount of the components with activity and based on the clinical common usability and the drug concentration similarity at present.

Based on this, both formulations (TBS 40 and TLI 40) were aseptically prepared under laminar air flow. Briefly, 2 mL of each gel was conditioned into a 5-mL polypropylene eyedropper, previously sterilized, and the dropper was closed with a polypropylene cap. Three batches of each formulation were prepared and subjected to a double filtration with 0.22-μm PES (polyethersulfone) filters.

The stability of each eyedrop was studied in unopened multidose eyedroppers for 4 months at three different temperature conditions: in refrigeration (4 ± 2 °C), at room temperature (25 ± 2 °C) and at oven temperature (40 ± 2 °C), protected from light exposure in all cases. Three units per formulation were subjected to tacrolimus quantification, osmolality determination, pH measurements and microbiological control growth at predetermined times (0, 15, 30, 45, 78 and 120 days). All samples were also visually inspected for any macroscopic changes (e.g., color, turbidity and precipitation).

#### 2.6.1. Quantification of Tacrolimus Amount

The determination of tacrolimus was performed as previously mentioned (see Section 2.1.1. Phase solubility diagrams). Refrigerated and accelerated samples were kept at room temperature for 20 minutes before quantification. Each sample was assayed in triplicate. The analytical method was validated according to International Conference on Harmonization (ICH) guideline recommendations [44].

#### 2.6.2. Osmolality, pH and Microbiological Control Growth

The pH and osmolality measurements were performed following the same experimental procedure previously described (see Section 2.4.1. pH and osmolality determination). In order to study the microbiological stability, 1 mL of each formulation was added in blood agar plates, Sabouraud/Chloranphenicol agar plates and liquid thioglycate medium plates. These samples were grown at 37 °C for predetermined periods (48 h, 15 days and 10 days, respectively). Once each incubation period ended, microbiological growth was observed and determined.

#### 2.6.3. Statistical Analysis 

Pharmaceutical Codex [58] has established the margins of the expiration period of the formulations as being once the concentration of active ingredient is reduced by 10% concerning the initial concentration. The percentage of unaltered drug versus time was fitted to a first order kinetics using GraphPad Prism^®^ 8 v.8.2.1 software and the degradation constant (*K*), expiration time (*t_90_*) and determination coefficient (*R^2^*) were calculated. pH and osmolality monitoring were performed to observe the presence of any changes. Microbiological stability was considered adequate when no microbial growth was provided in the cultured samples. The presence of any abnormal macroscopic particles in the formulations was not considered acceptable.

### 2.7. In Vivo Evaluation of the Residence Time on the Ocular Surface

In vivo studies were carried out on male Sprague-Dawley rats with an average weight of 250 g supplied by the animal facility at the University of Santiago Compostela (Spain). The animals were kept in individual cages under controlled temperature (22 ± 1 °C) and humidity (60 ± 5%), with day–night cycles regulated by artificial light (12/12 hours) and feeding ad libitum. The animals were treated according to the ARVO statement for the use of animals in ophthalmic and vision research as well as the approved guidelines for laboratory animals [59,60]. Experiments (idis12072017) were approved by the Institutional Committee of the Health Research Institute of Santiago de Compostela (IDIS) following the Galician Network Committee for Ethics Research, the Spanish and European Union (EU) rules (86/609/CEE, 2003/65/CE, 2010/63/EU, RD 1201/2005 and RD53/2013).

The positron emission tomography (PET) and computed tomography (CT) procedures for conducting the radiolabeling of the formulations and the quantitative ocular permanence study were described in our previous works [15,61,62]. Briefly, PET and CT images were acquired using the Albira PET/CT Preclinical Imaging System (Bruker Biospin^®^; Woodbridge, Connecticut, USA). Anesthetized animals (2.5% (*v/v*) isoflurane/oxygen) were positioned into the imaging bed, monitoring the respiration frequency. Afterward, 7.5 µL of each tacrolimus formulation, previously radiolabeled with ^18^F-fluorodeoxyglucose (^18^F-FDG), was instilled into the conjunctival sac of both eyes using a micropipette. The administered radioactivity was 0.20–0.25 MBq per eye. After the instillation, static PET frames at different times (0, 30, 75, 120, 240 and 300 min) were acquired as a way to evaluate the pharmacokinetics behavior. To prevent the rats from scratching their eyes by removing the instilled formulations, an Elizabethan collar was placed between PET studies. Two animals (4 eyes) were tested for each formulation in order to accomplish the 3Rs regulatory frameworks [63].

Image analysis was performed using the Amide´s Medical Image Data Analysis Tool [64]. Regions of interest (ROIs) were manually drawn for the different frames by delimiting the total radiotracer uptake of each eye using a spherical volume of 1767.1 m^3^ (15 × 15 × 15 mm). The radiotracer uptake over time was corrected by the radioisotope decay (^18^F half-life: 109.7 min). Subsequently, a clearance rate for each formulation was obtained in terms of the ocular remaining radioactivity uptake over time after instillation. The data were fitted using a non-compartmental analysis in order to calculate the elimination constant (*K*), the half-life (*t_1/2_*) and the zero and first moment pharmacokinetic parameters, area under curve (AUC_0_^∞^) and mean residence time (MRT) using GraphPad Prism^®^ 8 v.8.2.1 software.

## 3. Results and Discussion

### 3.1. Tacrolimus/HPβCD Solubilization Study

**Figure 3:**
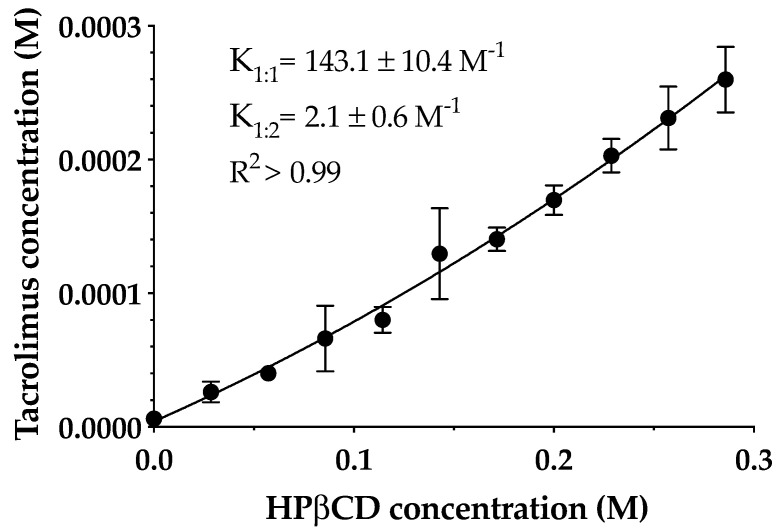
The phase solubility diagram of tacrolimus/hydroxypropyl-β-cyclodextrin (HPβCD) at 25 °C in purified water. Stability constants values represent the mean ± SD (*n* = 5).

The phase solubility diagram of tacrolimus in aqueous HPβCD solutions at 25 °C is shown in Figure 3. The resulting profile indicates an A_P_-type diagram that shows a positive deviation from characteristic linearity, this being supported by previous studies [27,37]. Thus, the formation of a soluble complex in the aqueous media with the formation of high-order drug/CD complexes at high cyclodextrin concentrations is assumed. It must be taken into account that the formation of 1:1 and 1:2 drug/CD complexes and both *K_1:1_* and *K_1:2_* stability constants were calculated. Tacrolimus aqueous solubility in the absence of cyclodextrins was 4 ± 0.67 μg/mL, and *K_1:1_* and *K_1:2_* values were 143.1 ± 10.3 and 2.1 ± 0.6 M^-1^, respectively.

The low values of stability constant indicate that tacrolimus and HPβCD interactions were weak, especially in the 1:2 complex. The main reason for this weak interaction between the drug and cyclodextrin may be the high molecular weight and the complex molecular structure of tacrolimus that hampered an adequate adaptation to the cyclodextrin cavity. This study was made only with HPβCD because according to the EMA, it is the safest cyclodextrin and most appropriated for topical ophthalmic administration together with sulfobutylether-β-cyclodextrin (SBEβCD), proving that it is not toxic [40]. This fact becomes important when transferring research to the clinic. Thus, NMR studies were performed in order to study the part of the drug and cyclodextrin structure involved in the complex formation.

**Figure 4:**
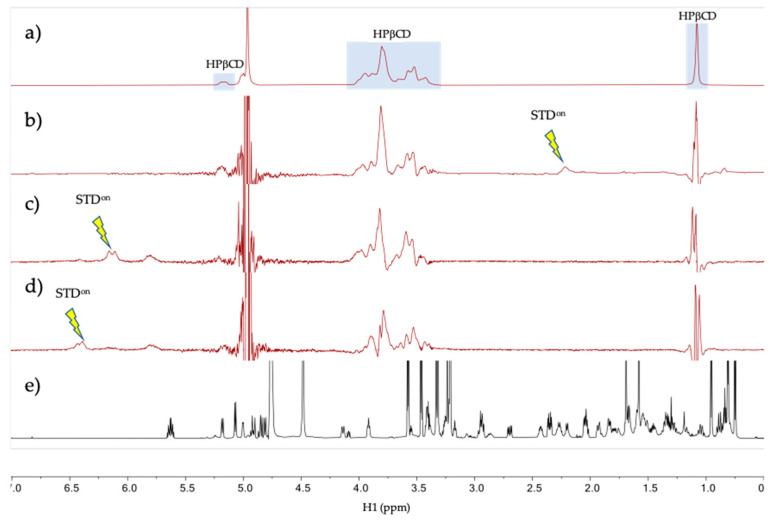
NMR spectra of tacrolimus/HPβCD interaction. (**a**–**d**) NMR spectra of the mixture tacrolimus/HPβCD in D_2_O at 278 K. (**a**) HPβCD ^1^H spectrum; (**b**) STD^on-off^ spectrum with on-irradiation at 2.12 ppm; (**c**) STD^on-off^ spectrum with on-irradiation at 6.12 ppm; (**d**) STD^on-off^ spectrum with on-irradiation at 6.30 ppm and (**e**) Reference ^1^H spectrum of pure tacrolimus in MeOD. The arrows indicate the approximate position of the on-saturation applied. Signals of HPβCD are indicated in spectrum (**a**).

The intermolecular interaction between tacrolimus and HPβCD in aqueous solution was studied by NMR in the tacrolimus/HPβCD mixture prepared in D_2_O at a 200 mM HPβCD concentration and tacrolimus saturation (0.2 mM). The ^1^H spectrum of the mixture at 278 K (Figure 4a) shows resonances at the chemical shifts expected for tacrolimus signals (a reference spectrum of pure tacrolimus in MeOD is given in Figure 4d) with a considerable broadening, a possible indication that the tacrolimus is forming a large aggregate or self-aggregate in the water solution due to its very low polar characteristics. To test for binding between the tacrolimus and HPβCD in the mixtures, a one-dimensional saturation transfer difference spectrum (STD) was used, being a well-known NMR technique for screening ligands binding to protein receptors [45,65]. STD spectra were measured with auto-subtraction of alternate scans acquired with off- and on-irradiation providing the so-called STD^on-off^ spectra [45]. In these STD spectra, the on-irradiation was placed in a certain proton signal of tacrolimus that is sufficiently separated in frequency from any HPβCD signal to prevent them being directly saturated. Three STD^on-off^ spectra were measured at 278 and 298 K under otherwise identical conditions. Interestingly, only the STD^on-off^ spectra measured at the lower temperature (278 K, but not at 298 K) provided STD signal responses for the HPβCD species, as can be seen in Figure 4b–d. This is due to the partial transfer of saturation from the tacrolimus signal being irradiated to the HPβCD resonances, which reflects that there is a binding equilibrium between these two molecules (Figure 4). The fact that the STD responses are only obtained in the spectra measured at 278 K and not at 298 K could indicate that there is low affinity between the two molecules at 298 K (i.e., binding equilibrium with Kd > 10 mM [66]), while the affinity is notably enhanced at 278 K (i.e., Kd < 1 mM [66]). Overall, these NMR results strongly suggest that tacrolimus is forming a large self-aggregate in the water solution and it is partially solubilized by forming a complex with HPβCD.

**Figure 5:**
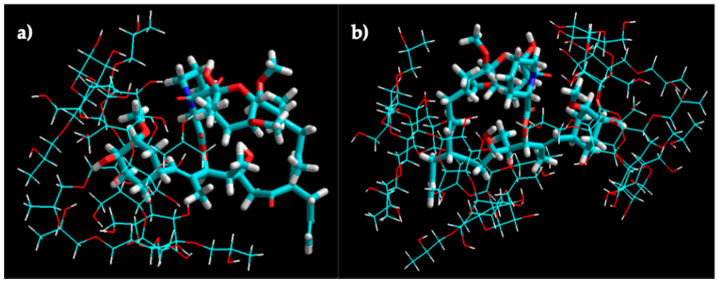
Molecular modeling of tacrolimus/HPβCD interaction. Molecular modeling of the 1:1 (**a**) and 1:2 (**b**) complex structures for tacrolimus and HPβCD obtained by manual docking and energy minimization using an MM+ force field in HyperChem^®^.

The molecular modeling of the tacrolimus/HPβCD interaction of 1:1 and 1:2 stoichiometry inclusion complexes is shown in Figure 5. The molecular docking studies indicated the higher probability of tacrolimus inclusion by its 4-hydroxy-3-methoxycyclohexyl group.

**Figure 6:**
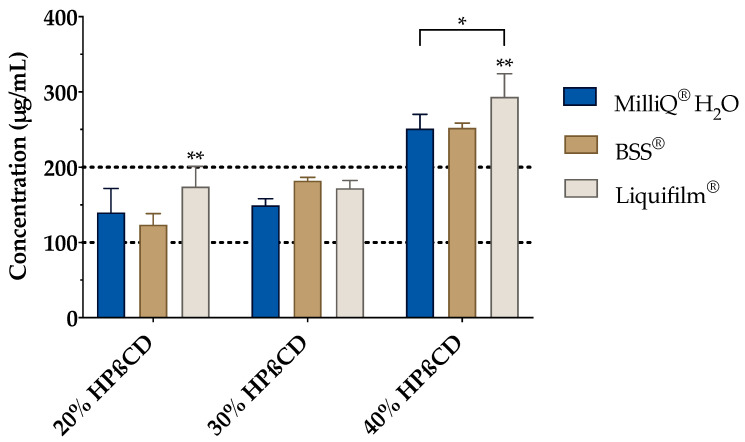
Tacrolimus solubility with 20%, 30% and 40% (*w/v*) of HPβCD in Balanced Salt Solution (BSS^®^) and Liquifilm^®^ vehicles. Statistical analysis: two-way ANOVA followed by Tukey´s multiple comparison test (* α < 0.05 compared with 20% (*w/v*) and 30% (*w/v*) HPβCD in all vehicles); one-way ANOVA followed by Tukey´s multiple comparison test (** α < 0.05 compared with the other two vehicles (MilliQ^®^ water and BSS^®^).

The effect of BSS and Liquifilm^®^ (as ophthalmic vehicles) on the complexation was also studied (Figure 6). Hence, a vehicle solubility study was experimentally carried out to determine the tacrolimus behavior with increasing HPβCD concentration media. As presented in Figure 6, a proportional increase in the tacrolimus solubility was observed by increasing the amount of cyclodextrin included into the vehicle. A two-way ANOVA was subsequently performed to compare all the media and assess the inclusion complexes’ formation. The resulting data showed no significant differences between the 20% (*w/v*) and 30% (*w/v*) HPβCD in all vehicles; nonetheless, statistically significant differences (α < 0.05) were found between 20% (*w/v*) and 30% (*w/v*) HPβCD solutions compared to the 40% (*w/v*) HPβCD solutions in all the vehicles. According to these results, the 30% (*w/v*) HPβCD formulations were discarded and only 20% (*w/v*) and 40% (*w/v*) HPβCD solutions were used in further studies.

The resulting data also showed significant differences (α < 0.05) among all the formulations for both HPβCD concentrations. Tukey´s multiple comparison test was then performed, and significant differences were also observed between Liquifilm^®^ and the other two vehicles (MilliQ^®^ water and BSS^®^) but there were none found between MilliQ^®^ water and BSS^®^. The tacrolimus solubility in Liquifilm^®^-diluted formulations may be attributed to the ternary complex formation (tacrolimus/HPβCD/PVA), increasing tacrolimus solubility by a synergistic solubilization effect [67].

**Figure 7:**
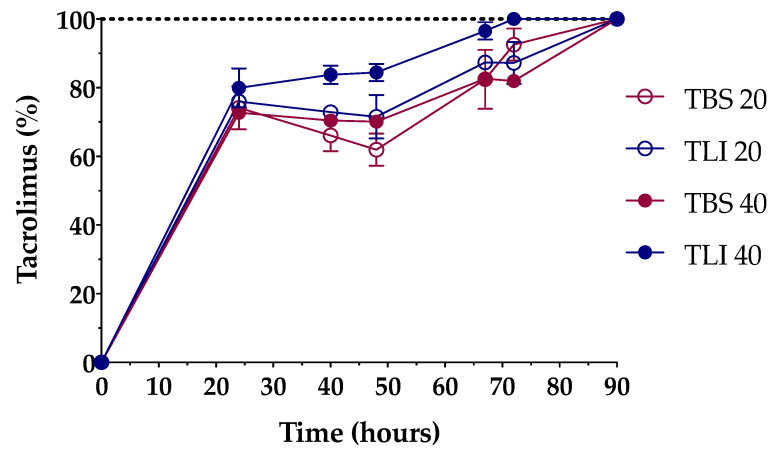
Dissolution time comparison among the different tacrolimus formulations. The 100% tacrolimus concentration corresponds to 0.01% (*w/v*) tacrolimus for TBS 20 and TLI 20 and 0.02% (*w/v*) tacrolimus for TBS 40 and TLI 40.

Formulations were successfully prepared by an inclusion complex/dissolution technique. Tacrolimus was incorporated into the HPβCD hydrophobic cavity, leading to an increase in its water solubility. The solubilization time was determined in order to know the time necessary to completely dissolve the required dose (Figure 7).

The time needed to prepare the aforementioned tacrolimus topical ophthalmic formulations will be a key preparation parameter in HPDs. As presented in Figure 7, 20% (*w/v*) HPβCD formulations containing tacrolimus reached 100% solubilization in a 90-h period, while 40% (*w/v*) HPβCD formulations containing tacrolimus showed different solubilization time, being quicker for the TLI 40 (72-h period, against the 90-h period for TBS 40). A two-way ANOVA was further carried out to study differences in tacrolimus solubilization time among the formulations. The resulting data showed statistically significant differences between TLI and TBS formulations (α < 0.05) from 40 h onwards. Nevertheless, no differences were found between TLI 20 and TLI 40, nor between TBS 20 and TBS 40.

### 3.2. Physicochemical Characterization

#### 3.2.1. pH, Osmolality and Surface Tension

pH and osmolality determinations were carried out in order to ensure that the formulations show values within the ocular physiological range. As presented in Table 2, all formulations met the pH and osmolality specifications described for topical ophthalmic administration. These results support the idea that these new formulations could be used without generating adverse effects in the ocular surface, compared to the control formulation (REF) [15] where, despite showing a pH value within the physiological range (pH 4 to 8) [68], the osmolality values (1220.5 mOsm/kg) were four times higher than the limit value for topical ophthalmic administration. Likewise, hypertonic topical ophthalmic formulations may alter the tear osmolarity and, thus, induce ocular inflammation, as described by Dutescu et al. [69].

Surface tension determination constitutes a key assay for a topical ophthalmic formulation. Appropriate surface tension values guarantee that a formulation may be spread evenly over the entire corneal surface, ensuring the drug optimal ocular penetration and also enhancing the comfort of the user when applying eye drops [70]. The ideal eye drop will have a surface tension similar to the tear film fluid (42–46 mN/m) [71]. Nonetheless, Han et al. previously studied the surface tension of commercialized ophthalmic formulations and the results showed that these topical ophthalmic solutions had surface tensions greater than that of tears (34.3–70.9 mN/m), most likely increasing the tear film stability and allowing for a greater lubrication of the eye [72].

Therefore, in the case of the designed formulations (TBS 20, TLI 20, TBS 40 and TLI 40), the surface tension values were slightly higher than physiological parameters, but they were still at an optimal value (see Table 2). These surface tension values can be given by HPβCD, which shows values of 54.8–57.5 mN/m in solution, as it was previously mentioned by Saokham et al. [67]. However, the reference formulation (REF) showed lower surface tension values than the others, being associated to the presence of ethanol in its composition.

A one-way ANOVA was applied to determine the surface tension of each formulation, and statistically significant differences were observed (α < 0.05). Tukey’s multiple comparison test was also performed, and no statistically significant differences were found between the TLI 20 and TLI 40 formulations, but significant differences were observed between the rest of the formulations (α < 0.05).

#### 3.2.2. Squeezing Force Determinations

**Figure 8:**
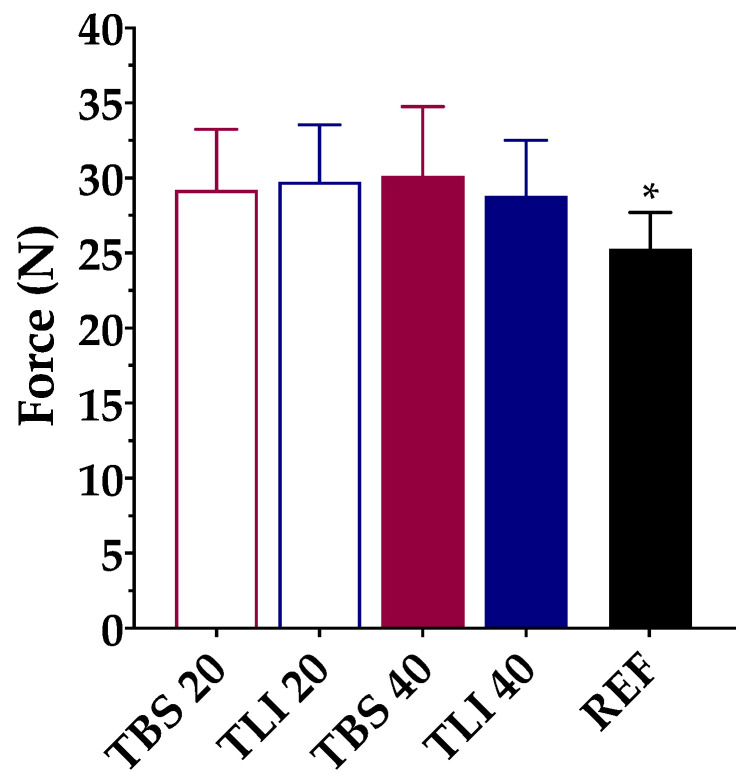
Comparison of squeeze force (N) values among different tacrolimus formulations (TBS 20, TLI 20, TBS 40, TLI 40 and REF). Statistical analysis: one-way ANOVA followed by Tukey´s multiple comparison test (* α < 0.05 compared with prepared formulations).

Figure 8 show the squeezing force test results for the studied formulations. Based on the research of Conner et al. [73], a large percentage of patients (>50%) receiving local ophthalmic treatments reported that self-management is difficult due to the need to apply force to the eyedropper in order to get the preparation out. Besides, high doses may cause the patient to suffer from side effects, while underdosing may cause damage or prolonged drug therapy. The volume and structure uniformity of the formulation drops are also important properties to ensure accurate drug dosage and avoid treatment variability.

The squeezing force test may be affected by different factors such as the formulation viscosity, surface tension or dropper tip design [74]. Therefore, the same type of packaging for testing was used. A one-way ANOVA was applied to determine the required force to dispense a drop of each formulation, and statistically significant differences were observed (α < 0.05). Tukey’s multiple comparison test was also applied, and no statistically significant differences were found between the tacrolimus/HPβCD formulations, but significant differences were observed between the reference formulation (REF) and the prepared formulations (α < 0.05).

### 3.3. Corneal Mucoadhesion

**Figure 9:**
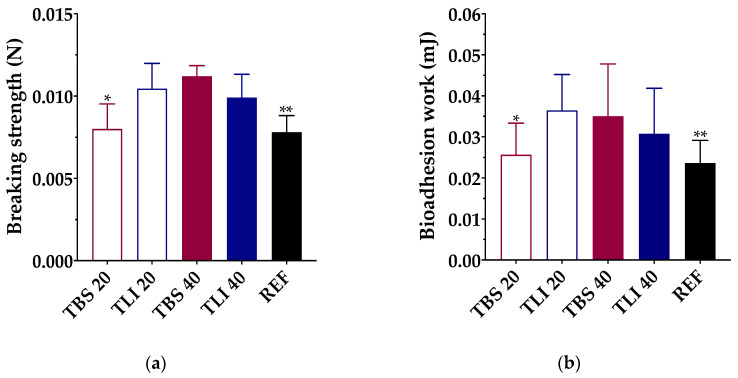
(**a**) Maximum breaking strength (N) and **(b)** bioadhesion work (mJ) obtained for each formulation using bovine cornea as a substrate. Statistical analysis: (**a**) one-way ANOVA followed by Tukey´s multiple comparison test (* α < 0.05 compared with TLI 20, TBS 40 and TLI 40 and ** α < 0.05 compared with TLI 20, TBS 40 and TLI 40); (**b**) one-way ANOVA followed by Tukey´s multiple comparison test (* α < 0.05 compared with TLI 20 and ** α < 0.05 compared with TLI 20 and TBS 40).

Knowing that the mucoadhesion properties of the topical ophthalmic formulations will give an approximate idea of the permanence time on the ocular surface, bioadhesion work measurements were performed with the studied topical ophthalmic formulations. All data were assessed through breaking strength (N) and bioadhesion work (mJ) measurements (Figure 9).

A one-way ANOVA was performed to define the bioadhesion work of the studied formulations, and statistically significant differences were observed (α < 0.05). Tukey’s multiple comparison test was also applied, and statistically significant differences were found between the TBS 20 and TLI 20 formulations and between the REF and TLI 20 and TBS 40, but no significant differences were observed between the rest of the formulations (α > 0.05).

The highest bioadhesion work values were obtained for the TLI 20 (0.036 ± 0.009 mJ) and TBS 40 (0.035 ± 0.013 mJ) formulations, and the lowest values were obtained for TBS 20 (0.026 ± 0.008 mJ) and REF (0.023 ± 0.005 mJ). In the case of TLI 40 (0.031 ± 0.011 mJ), no statistically significant differences were observed compared with TLI 20 and TBS 40. It was observed that during the separation stage of the cornea from the formulation, a formulation film remained adhered to the cornea when the load cell was lifted. It can be assumed, then, that the formulation interacts with the corneal surface with more intensity than the own cohesive forces of the formulation. Therefore, the adhesive bond fails due to the formulation fracture, making the viscosity and consistency play a fundamental role.

An in vivo ocular permanence study was subsequently performed using a PET/CT imaging technique to confirm the bioadhesive behavior of the formulations.

### 3.4. Ocular Irritancy and Toxicity

Some eye irritation and toxicity assessment tests have shown considerable potential to eliminate procedures that were historically performed by animal experimentation, such as the Draize rabbit eye irritation test which presents one of the most criticized and contested animal tests [75]. The efficacy of these in vitro and ex vivo procedures has been well studied by pharmaceutical industries and some national regulatory agencies [76]. The replacement of the Draize rabbit eye test with alternative models includes physicochemical tests, cell and tissue culture systems or organotypic models as eye components or isolated eyes [76,77,78].

The strategy to replace the Draize test by combining several animal-free methods has raised expectations [75]. The combination of two different methods has been proposed in this work. The Bovine Corneal Opacity and Permeability test (BCOP), which allows to detect whether the tested compounds cause a moderate, severe or very severe irritation, combined with the hen’s egg test on the chorioallantoic membrane assay (HETCAM) covers the whole spectrum of irritation, since mild or very mild irritation signs can be detected.

#### 3.4.1. Bovine Corneal Opacity and Permeability Test (BCOP)

**Figure 10:**
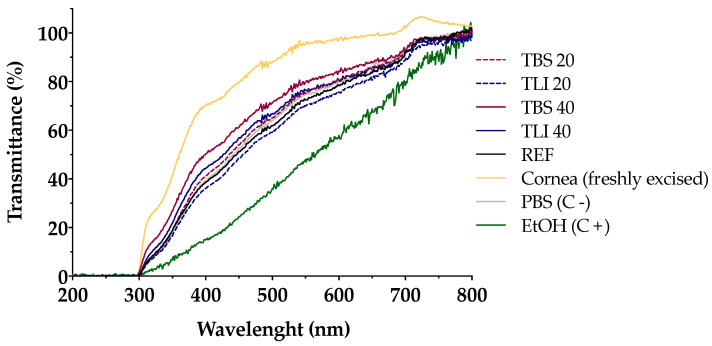
Ultraviolet–visible (UV–Vis) scan (from 200 to 800 nm) of corneal transmittance (%) values of bovine corneas treated with TBS 20, TLI 20, TBS 40, TLI 40, REF, PBS (negative control) and ethanol (positive control) after 10 min tacrolimus formulation treatment and 120 min PBS treatment.

**Figure 11:**
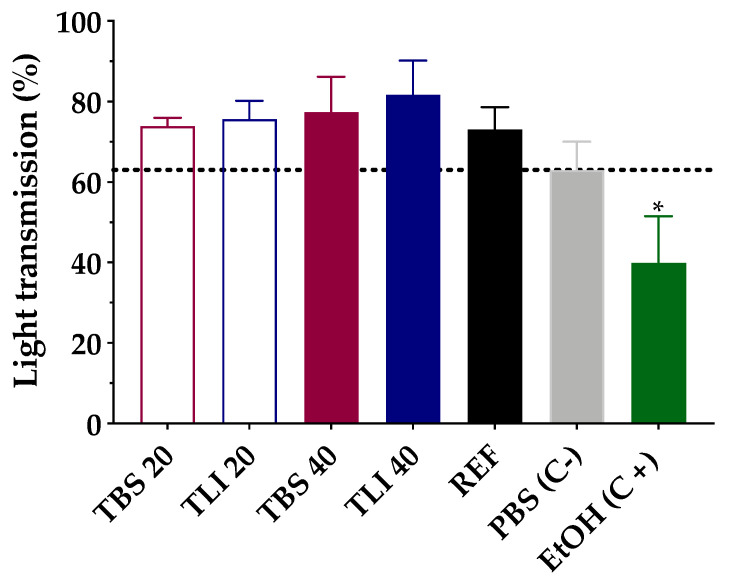
Opacity values of bovine corneas treated with TBS 20, TLI 20, TBS 40, TLI 40, REF, PBS (negative control) and ethanol (positive control) after 10 min tacrolimus formulation treatment and 120 min PBS treatment. Here, 63% light transmission corresponds to the total light transmitted through bovine corneas incubated in PBS. Statistical analysis: one-way ANOVA followed by Tukey´s multiple comparison test (* α < 0.05 compared with prepared formulations, reference formulation and negative control).

Transmittance values of opacity and permeability assays were studied to assess whether formulations induce corrosivity or irritation (see Figure 10 and Figure 11). No significant structural changes were observed in terms of corneal opacity and fluorescein permeability when comparing all formulations to the negative control solution (PBS) but were observed with regard to the positive control (ethanol) (α < 0.05).

Similarly, opacity and permeability data were corrected for background or control values prior to further statistical determinations being estimated. The IVIS score was then calculated and all formulations resulted in an in vitro irritation score of 0 (IVIS = 0), showing no toxic effects compared to control formulations.

Likewise, the fluorescein permeability test was further applied on the corneas previously treated with the tested formulations. The resulting data showed no statistically significant differences between all the formulations; however, significant differences (α < 0.05) were observed between the studied formulation and the positive control (ethanol).

#### 3.4.2. Hen’s Egg Test on the Chorioallantoic Membrane (HETCAM)

**Figure 12:**
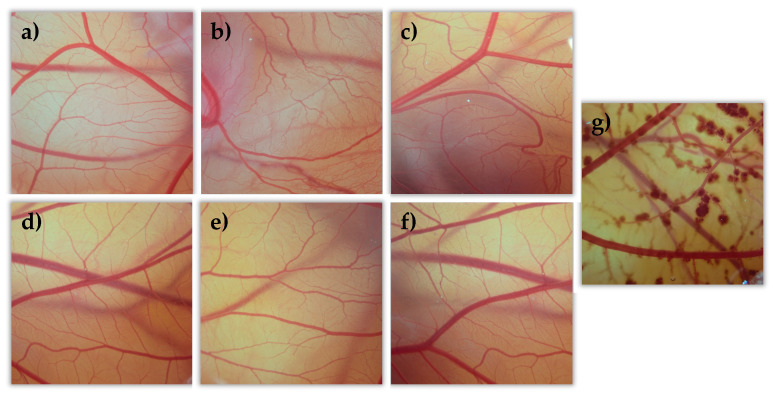
Hen’s egg test on the chorioallantoic membrane (HETCAM) images 5 min post-instillation for the different formulations. (**a**) NaCl (C-); (**b**) TBS 20; (**c**) TLI 20; (**d**) TBS 40; (**e**) TLI 40; (**f**): REF; (**g**) NaOH (C+).

All tacrolimus eyedrops were tested on the egg’s CAM as well as two controls, NaCl as a negative control (C-) and NaOH as a positive control (C+). All formulations were evaluated, and data were compared to the NaCl and NaOH solutions’ results. All formulations showed no toxic effects (irritation score = 0) compared them with the positive control formulation (see Figure 12). This agrees with previously described BCOP test results.

### 3.5. Stability Study

**Figure 13:**
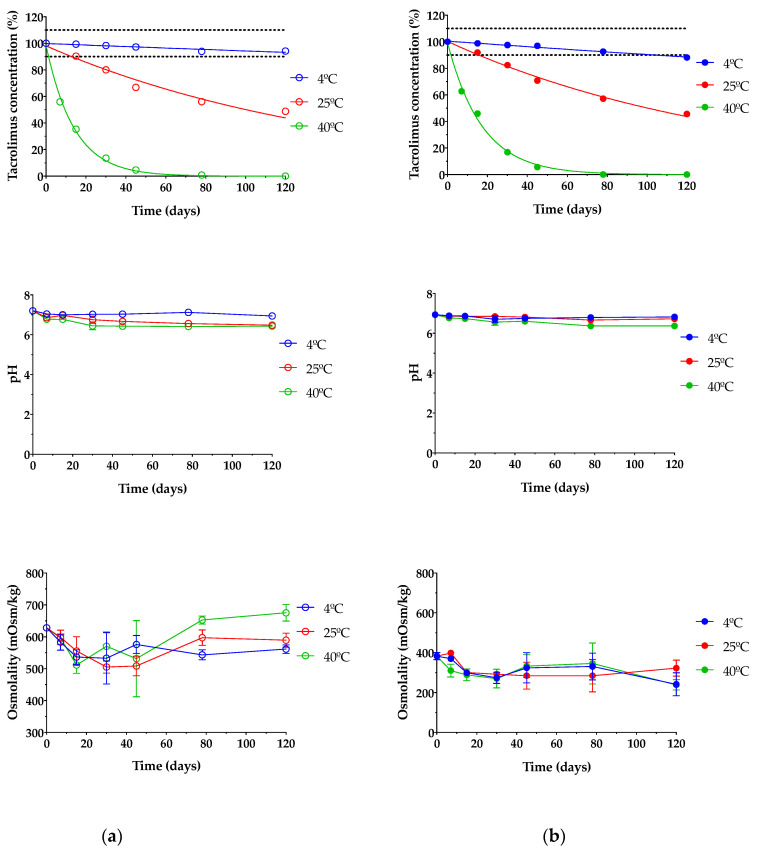
Tacrolimus concentration, pH and osmolality stability of (**a**) TBS 40 and (**b**) TLI 40 stored under three different temperature conditions: in refrigeration (4 ± 2 °C), at room temperature (25 ± 2 °C) and at oven temperature (40 ± 2 °C) during a 4-month stability test.

Stability studies of pharmaceutical compounds are essential to ensure drug efficacy [79,80] as well as to know if degradation products can cause toxic side effects and other undesired effects [27]. In this assay, only TBS 40 and TLI 40 were studied due to all formulations containing the exact same qualitative composition; thus, the formulations with the highest tacrolimus concentrations were tested as representative; these formulations were chosen based on the clinical common usability and the drug concentration similarity at present. As presented in Figure 13, it can be seen that temperature was an important factor in the stability under storage of tacrolimus/HPβCD ophthalmic formulations. TBS 40 and TLI 40 showed the same tacrolimus degradation pattern for each storage condition and no statistically significant differences were found between them (α > 0.05).

Table 3 shows the degradation constant (*K*), expiration time (*t_90_*) and determination coefficient (*R^2^*) values for both tacrolimus eye drops. It must be taken into account that TBS 40 showed a higher degradation rate (1.23 times) than TLI 40 for the oven temperature condition, though significant degradation rate variations were not observed for the other two storage conditions.

Eye drops stored at oven temperature (40 ± 2 °C) were not stable in the first 7 days of the study, observing a rapid tacrolimus degradation process. In the case of eye drops kept at room temperature (25 ± 2 °C), the tacrolimus degradation was not so abrupt, but from day 15 of the study, the tacrolimus concentration was reduced to below 90% of the initial concentration, so the formulation was no longer stable. Nonetheless, both eye drops preserved in refrigeration (4 ± 2 °C) were stable beyond the 3-month period; specifically, the initial concentration of tacrolimus previously fell below 90% in the TLI 40 formulation compared to the TBS 40 formulation. Therefore, the optimal storage condition for tacrolimus topical ophthalmic formulations was at 4 °C.

In this way, it was determined that eye drops kept in refrigeration condition have an available period of at least 3 months. In the case of TLI 40, the presence of benzalkonium chloride may be beneficial to the stability of tacrolimus once opened by minimizing microbial contamination.

Prajapati et al. studied tacrolimus degradation in aqueous HPβCD solutions [27]. Their results showed an inversely proportional correlation between tacrolimus degradation rate and HPβCD concentration values, with a maximum degradation value where no HPβCD was included into the formulations. The presence of 40% (*w/v*) HPβCD in the formulations used in the present study suggested that the increased stability of tacrolimus was due to the inclusion of complex formation between HPβCD and tacrolimus.

Likewise, pH and osmolality measurements showed no significant changes over the course of the study regardless of storage condition (α > 0.05). The results guaranteed that all tested formulations were also in the appropriate range for topical ophthalmic administration, ensuring suitability for an accurate tacrolimus ocular penetration.

Furthermore, microbiological control growth is a test to take into account the quality control of the prepared formulations since it ensures the sterility of the preparations and, therefore, the conditions of asepsis in the production process. Sterility is one of the most important requirements when preparing ophthalmic formulations as it reduces the risk of eye infections. In this study, an adequate conservation of the eye drops was observed for each condition since no presence of microorganisms was found in any of the studied samples. In addition, no macroscopic changes (e.g., color, turbidity and precipitation) were observed during the whole study period. Thus, the absence of microorganisms and suspended particles was in good agreement with the preparation of this type of topical ophthalmic formulations in HPDs, by a simple technique with non-strict equipment requirements.

### 3.6. In Vivo Evaluation of the Residence Time on the Ocular Surface

The development of new ophthalmic topical vehicles for increasing drugs’ permanence on the ocular surface is important to improve drug bioavailability in the eye as well as the treatment adherence by patients [81]. PET/CT imaging is a relatively new imaging modality that provides a quantifiable signal on the pharmacokinetic profile of the topically administered radiopharmaceutical. In this way, it allows to obtain a signal in the eye after an eye drop instillation and its subsequent follow-up at different study times, and to be able to follow the biodistribution of the formulation in the nasolacrimal duct and nasal cavity [62].

In this study, the PET/CT imaging technique was used to determine the residence times of the proposed tacrolimus topical ophthalmic formulations on the ocular surface, and they were compared with the tacrolimus eye drop (REF) elaborated by HPDs.

**Figure 14:**
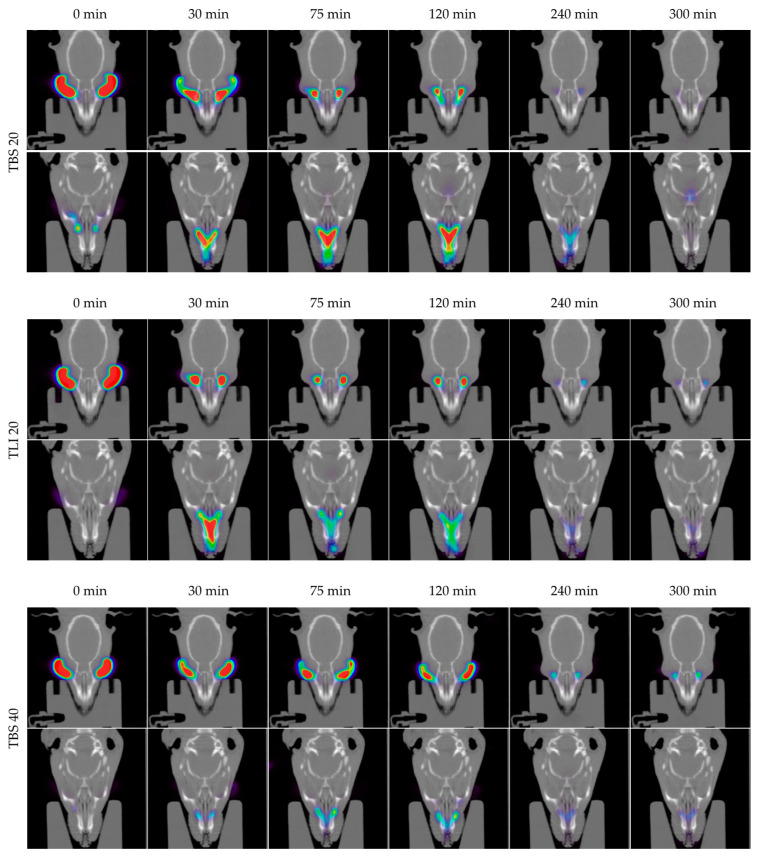

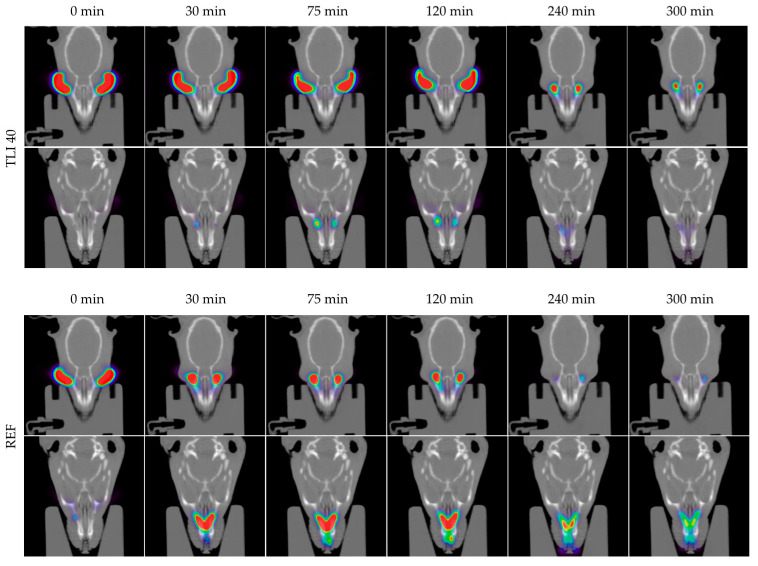
Fused positron emission tomography (PET)/computed tomography (CT) images displayed in coronal plane representing rat eyes (above) and nasolacrimal duct (below) with TBS 20, TLI 20, TBS 40, TLI 40 and REF at 0, 30, 75, 120, 240 and 300 minutes post-administration. The formulation signal on the eye surface is coded on a color scale: blue areas show low radioactive activity whereas red areas show high radioactive activity.

In Figure 14, the permanence of the studied eye drops at different times can be seen more visually, with PET/CT images showing both coronal planes (rat eyes and nasolacrimal duct).

**Figure 15:**
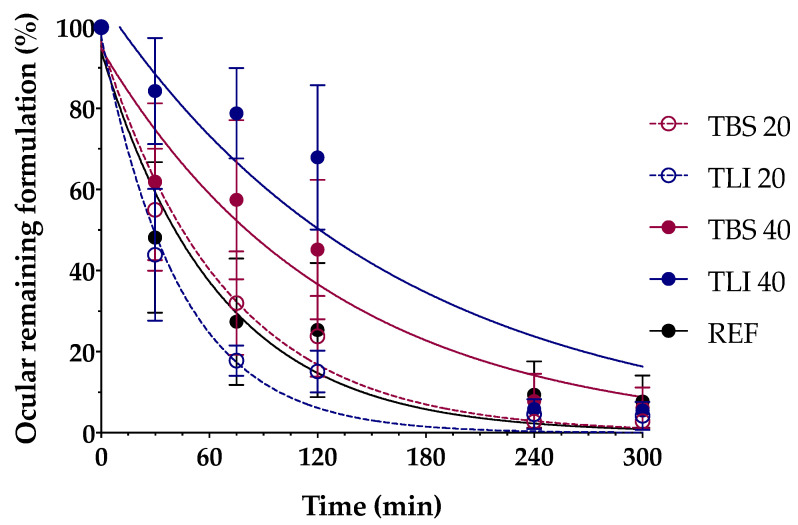
Tacrolimus eyedrop clearance rate (TBS 20, TLI 20, TBS 40, TLI 40 and REF) from the ocular surface determined by PET. Resulting data are represented in eye remaining radioactivity uptake (%) vs time after instillation.

Figure 15 shows the clearance rate for each tested formulation compared to the tacrolimus eye drop (REF) elaborated by HPDs. The clearance rate was represented in terms of the ocular remaining radioactivity uptake over time after instillation and the corresponding fits in order to estimate all pharmacokinetic parameters (*K*, *t_1/2_*, AUC_0_^∞^ and MRT) and the remaining formulation at 75 min (%), which are represented in Table 4. All the parameters indicated a significant increase in the ocular retention time for the TBS 40 and TLI 40 formulations compared to TBS 20 and TLI 20; the best formulation result was obtained for TLI 40. The results from the ocular permanence assays showed that formulations containing higher concentrations of HPβCD had a longer eye residence time, regardless of the vehicle used. Adding HPβCD to formulations, apart from increasing the solubility and stability of tacrolimus, also gave a more viscous and stickier characteristic to the solution. This feature helps the eye drop to spread and be more retained on the eye surface as it can be seen during eye drop instillation. PET/CT studies showed that the formulations were mucoadhesive and had an adequate consistency to remain on the ocular surface for a long time.

A two-way ANOVA was applied to the % of remaining formulation on the ocular surface parameter in order to evaluate the influence of the time and the formulation in its clearance to determine whether there were differences in the permanence of the formulations on the corneal surface. No statistically significant differences were observed between 20% (*w/v*) HPβCD formulations compared to REF. Nevertheless, statistically significant differences were observed (α < 0.05) between 20% (*w/v*) HPβCD formulations and 40% (*w/v*) HPβCD formulations as well as between 40% (*w/v*) HPβCD formulations and REF for times beyond 30 min post-administration.

The TLI 40 vehicle (Liquifilm^®^) is composed of PVA; this polymer has a mechanism based on the interdiffusion of polymer chains across the bioadhesive interface that produces entanglements and physical bonds between the polymer and the substrate. The intimate contact and the presence of hydroxyl radicals in the polymer can promote the establishment of weak interactions with the mucin layer (i.e., hydrogen bonds) [82]. The mucoadhesion study assumptions related to TLI 40 formulation were confirmed by the PET/CT imaging technique.

The conventional pharmacological treatments for uveitis are associated with strict patient compliance, limited efficacy due to the appearance of refractory processes and different severe side effects. HPDs have resorted to a reformulation process of intravenous formulations as a way to obtain new topical ophthalmic pharmacy compounding as a pharmacological alternative. One of the most used pharmacy compounding products was based on Prograf^®^ reformulation, this being an hydroalcoholic eye drop containing 0.03% (*w/v*) tacrolimus. Nevertheless, this type of treatment has shown different disadvantages, including high osmolality and patient discomfort due to the eye drop composition.

Different formulations are proposed in the present work as new pharmacological alternatives, specifically intended for topical ophthalmic administration for uveitis treatment. These formulations have proven to be adequate for this administration pathway, showing several advantages compared to the currently used treatments, including I) patient comfort improvement, II) great efficacy with a simple preparation method, III) easy translational research to HPDs and IV) a health expenditure reduction in uveitis treatment.

## 4. Conclusions

In this work, a consistent tacrolimus solubilization study was carried out as a way to deeply understand this drug behavior in future topical ophthalmic formulations. Tacrolimus/HPβCD interaction in solution was confirmed by phase solubility diagram, NMR and molecular modeling studies, and the influence of the vehicle was also studied. The use of 40% (*w/v*) HPβCD allowed to prepare eye drop solutions with a 0.02% (*w/v*) tacrolimus concentration that could be in the therapeutic range for uveitis treatment.

The developed HPβCD-based formulations showed pH, osmolality, surface tension and safety values in the optimum range for topical ophthalmic administration. Stability studies showed no changes in the eye drops kept in refrigeration condition for at least 3 months, which could facilitate the preparation programming and improve the patient comfort.

Additionally, ex vivo mucoadhesion and in vivo ocular permanence studies showed good mucoadhesive properties and lower ocular clearance for TBS 40 and TLI 40, almost doubling the permanence half-life time in the ocular surface compared to the REF pharmacy compounding.

Taking into account all of the obtained results, TLI 40 was proposed as the best candidate. This eye drop allowed reaching a 0.02% (*w/v*) drug dose, which was safe and showed the best mucoadhesive and ocular permanence properties. In addition, the presence of benzalkonium chloride in Liquifilm^®^ could help to prevent microbial growth once the eye drops were opened.

## Figures and Tables

**Table 1:** Tacrolimus eye drops composition.

Formulation	Composition		
	HPβCD (*w/v*, %)	Tacrolimus (*w/v*, %)	Vehicles
TBS 20	20	0.01	BSS^®^
TLI 20	20	0.01	Liquifilm^®^
TBS 40	40	0.02	BSS^®^
TLI 40	40	0.02	Liquifilm^®^
REF	-	0.03	Liquifilm^®^

**Table 2:** pH, osmolality and surface tension results of tacrolimus/HPβCD formulations.

Formulations	pH		Osmolality (mOsm/kg)		Surface Tension (mN/m)	
	Mean	SD	Mean	SD	Mean	SD
TBS 20	7.036	0.021	359.3	5.86	54.63	0.93
TLI 20	6.986	0.005	283.6	2.52	58.46	1.10
TBS 40	7.203	0.005	628	6.93	51.5	0.61
TLI 40	6.933	0.005	383	18.08	58.43	0.21
REF	7.3	0.014	1220.5	18.57	47.5	0.2

**Table 3:** The degradation constant *K*, *t_90_* and *R^2^* of two tacrolimus eye drops (TBS 40 and TLI 40) obtained by interpolation of the calculated regression line (% of remaining tacrolimus concentration vs time).

Formulations	Storage Condition	*K* (days^−1^)		*t_90_* (days)		*R^2^*	
		Mean	SD	Mean	SD	Mean	SD
TBS 40	Refrigeration (4 °C)	0.0006	4.3 × 10^−5^	184.32	13.5	0.8890	0.0179
	Room temperature (25 °C)	0.0067	0.0002	15.72	0.53	0.9645	0.0057
	Oven temperature (40 °C)	0.0713	0.0016	1.48	0.03	0.9966	0.0012
TLI 40	Refrigeration (4 °C)	0.0011	5.8 × 10^−5^	98.50	5.2	0.9410	0.040
	Room temperature (25 °C)	0.0067	0.0002	15.66	0.40	0.9867	0.0035
	Oven temperature (40 °C)	0.0577	0.0004	1.82	0.01	0.9959	0.0007

**Table 4:** Pharmacokinetics parameters (*K*, *t_1/2_*, AUC_0_^∞^, mean residence time (MRT) and % remaining formulation at 75 min) of the tacrolimus eye drops obtained by the fitting of the percentage formulation remaining on ocular surface.

Formulations	*K* (min^−1^)		*t_1/2_* (min)		AUC_0_^∞^ (% × min)		MRT (min)		Remaining Formulation at 75 min (%)	
	Mean	SD	Mean	SD	Mean	SD	Mean	SD	Mean	SD
TBS 20	0.014	0.003	49.70	10.34	74.96	7.70	72.90	6.02	31.99	12.79
TLI 20	0.025	0.008	29.40	8.51	59.16	11.18	73.94	22.62	17.79	3.74
TBS 40	0.024	0.023	61.79	46.16	89.61	57.41	76.43	35.70	57.46	19.65
TLI 40	0.011	0.007	86.22	38.93	123.31	51.18	90.51	17.67	78.82	11.15
REF	0.018	0.010	46.34	22.88	82.22	30.2	93.28	37.55	27.44	15.65

## Data Availability

Not applicable.
